# Zr^4+^-Doped Anatase TiO_2_ Nanotube Array Electrode for Electrocatalytic Reduction of L-cystine

**DOI:** 10.3390/ma13163572

**Published:** 2020-08-13

**Authors:** Weizhen Wang, Guangxin Wang, Xuhui Zhao, Xiaofeng Zhang, Yuming Tang, Yu Zuo

**Affiliations:** Beijing Key Laboratory of Electrochemical Process and Technology for Materials, Beijing University of Chemical Technology, Beijing 100029, China; 13260335510@163.com (W.W.); 2018200513@mail.buct.edu.cn (G.W.); zhangxf@mail.buct.edu.cn (X.Z.); zuoy@mail.buct.edu.cn (Y.Z.)

**Keywords:** nanocomposites, oxidation, titanium dioxide, electrocatalysis

## Abstract

A Zr^4+^-doped anatase TiO_2_ nanotube array electrode was prepared using a process that included Ti anodizing, chemical immersion, and heat treatment. The compositions, microstructure, and electrochemical properties of the prepared electrodes were characterized. The results show that Zr^4+^ was successfully introduced into the TiO_2_ nanotube array electrodes. Because Zr^4+^ was doped into the crystal structure of the TiO_2_and replaced a part of Ti^4+^ to form more oxygen vacancies and Ti^3+^, the electrocatalytic activity of the prepared electrodes, for the reduction of L-cystine, was significantly improved.

## 1. Introduction

L-cysteine is widely used in many fields, such as medicine, cosmetics, and biochemical research. The typical industrial production of L-cysteine is achieved through the electrocatalytic reduction of L-cystine. The currently used Pb electrodes, or other catalytic electrodes with deposited Pb, are prone to heavy metal pollution in acid electrolytes. Although titanium electrodes have also been used in the reduction of L-cystine, the effect is not satisfactory [1]. Therefore, developing alternative materials with stable performance, that are environmentally friendly and have a high catalytic reduction activity, are one of the current research hotspots [2,3,4].

As one of the most studied catalytic materials, TiO_2_ has an important role in the field of catalysis [5,6,7,8,9,10,11]. Skúlason et al. [12] discussed the role of transition metal oxides in the electrocatalytic reduction of N_2_ by using density functional theory (DFT) calculations. Hirakawa et al. [13] reported the role of oxygen vacancies and Ti^3+^ in TiO_2_ in the photocatalytic reduction of N_2_. In order to enhance the catalytic activity of TiO_2_, doping metal elements are used to increase the vacancies and defects in the TiO_2_ crystal structure [14,15,16,17]. At present, most of the correlative research in this field mainly focuses on the photocatalysis of TiO_2_. However, there are relatively few studies on its electrocatalysis, especially regarding electrocatalytic reduction. Recently, Cao et al. [18,19] reported in detail that a Zr^4+^-doped TiO_2_ electrode can efficiently reduce N_2_ through electrocatalysis. This provides a feasible idea from which we can design a TiO_2_ nanotube array electrode with a high electrocatalytic reduction activity for reducing L-cystine. Moreover, considering the better stability of titanium and the existence of the oxygen vacancies and Ti^3+^ in the anatase TiO_2_, the TiO_2_ nanotube array electrode might also possess good potential in the field of electrocatalytic reduction.

We have designed a Zr^4+^-doped anatase TiO_2_ nanotube array electrode (anatase Zr/TiO_2_), in which Zr^4+^ partly replaces Ti^4+^ in the anatase TiO_2_, and studied its electrocatalytic reduction activity for reducing L-cystine and discussed its reduction mechanism.

## 2. Materials and Methods

**Figure 1:**
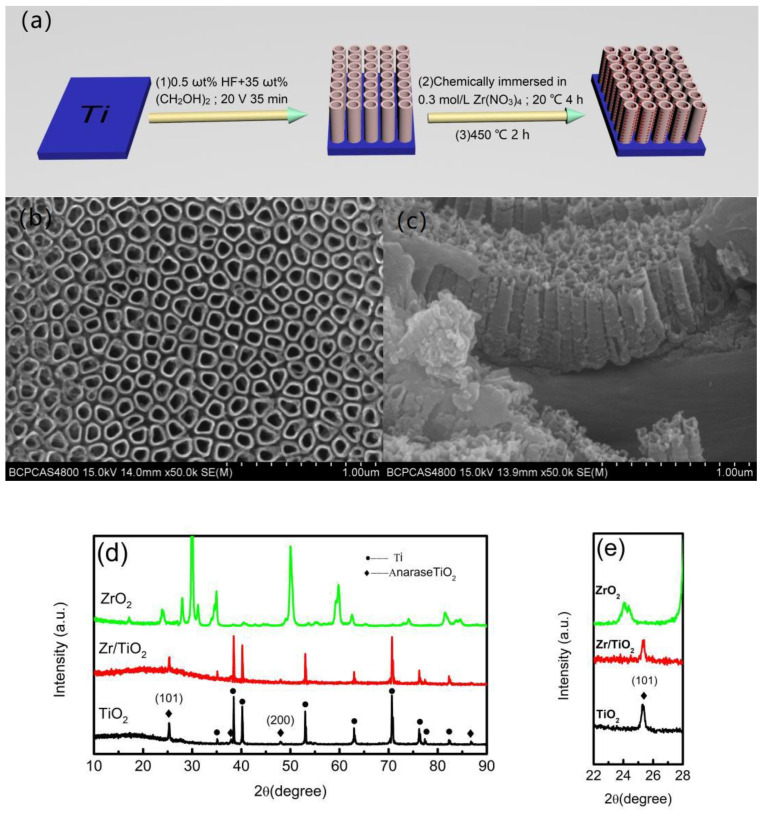
Preparation process (**a**), surface (**b**), and section (**c**) morphologies, and XRD patterns (**d**,**e**) of anatase Zr/TiO_2_ nanotube array electrode.

The preparation process for the anatase Zr/TiO_2_ electrode is shown in Figure 1a. Firstly, the TiO_2_ nanotube arrays on the pure Ti foil (99.99 wt%) surface was prepared through anodizing, which was carried out in 35 wt% (CH_2_OH)_2_ (ethylene glycol) + 0.5 wt% HF (hydrofluoric acid) solutions, under a constant voltage of 20 V for 35 min at room temperature. The auxiliary electrode was a graphite electrode. After anodization, the samples were soaked in deionized water and then chemically immersed in a 0.3 mol·L^−1^ Zr(NO_3_)_4_ solution for 4 h, in order to dope Zr^4+^. Subsequently, the samples were washed with deionized water and ethanol, several times. Finally, they were heated to 450 °C, kept for two hours, and cooled slowly in a muffle furnace.

The crystal structure of the modified electrode surface was studied using X-ray diffraction (XRD) (Bruker D8 advance, Cu Kα, λ = 0.1548 nm, Berlin, Germany). The morphology, length, and diameter of the TiO_2_ nanotubes on the electrode surface were characterized using a SEM (S-4800, Hitachi, Tokyo, Japan). The existence and valence of Ti and Zr on the surface of the Zr/TiO_2_ electrode were characterized using X-ray photoelectron spectroscopy (XPS) (PHI 1600 ESCA, PerkinElmer, Waltham, MA, USA). The binding energies of the peaks were calibrated using the binding energy of the C1s peak (285 eV).

The electrochemical performance was tested using the electrochemical workstation (CS350H, Wuhan Corrtest, Wuhan, China). In the three-electrode system, the auxiliary electrode was a Pt electrode, and the reference electrode was a saturated calomel electrode. The test solutions were HCl solutions containing L-cystine.

## 3. Results

Scanning electron microscopy (SEM) images show that the anatase Zr/TiO_2_ electrode has a tubular structure, with tube diameters and lengths of about 100 and 650 nm, respectively (Figure 1b,c). The crystalline structures of the different samples were studied using X-ray diffraction (Figure 1d)—for both the undoped and Zr^4+^-doped TiO_2_ nanotube arrays. The other diffraction peaks correspond to the anatase phase (JCPDS # 21‒1272). A close examination of the pattern (Figure 1e), after doping Zr^4+^, revealed that the peak intensity of the TiO_2_ slightly decreased. According to the Scherrer equation, the calculated grain sizes of the TiO_2_ (101) were about 7 and 5.1 nm for the undoped and doped samples, respectively, suggesting that the grain sizes of the TiO_2_ also became slightly smaller after doping Zr^4+^. Above, the results indicate that the crystallinity of the TiO_2_ slightly decreased. No diffraction peak relating to the ZrO_2_ was observed in the XRD pattern (JCPDS # 79-1768). Compared to Ti^4+^, Zr^4+^ is suitable in size, and is similar in d electron configuration and oxide structure (Zr^4+^ 72 pm, Ti^4+^ 52 pm) [15]. Zr^4+^ was doped into the anatase TiO_2_ to replace a part of Ti^4+^, and did not change the anatase crystal structure [18].

**Figure 2:**
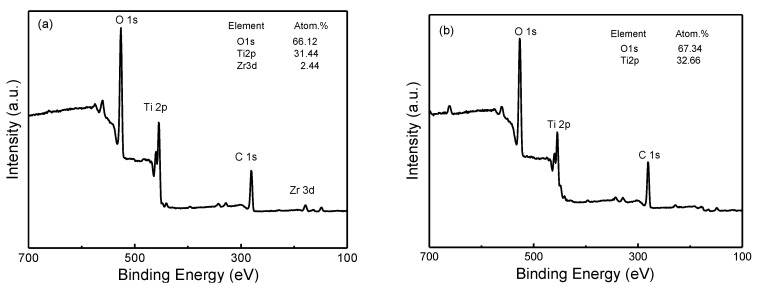
XPS spectra of TiO_2_ nanotube array surface: (**a**) Zr^4+^ doped; (**b**) undoped.

**Figure 3:**
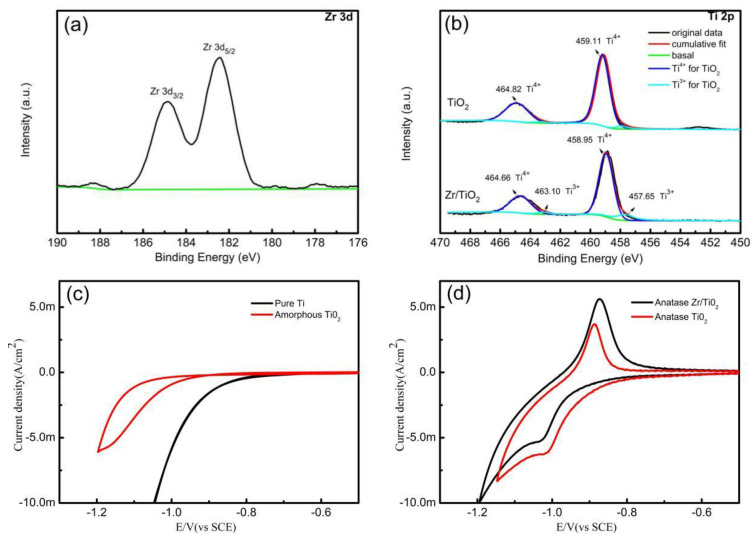
XPS spectra of undoped and Zr^4+^ doped TiO_2_ nanotube array surface: (**a**) Zr 3d, (**b**) Ti 2p; CVs of different electrodes in 2 mol·L^−1^ HCl solution (scanning rate: 20 mV·s^−1^): (**c**) pure titanium and amorphous TiO_2_ nanotube array electrode; (**d**) undoped and Zr^4+^ doped anatase TiO_2_ nanotube array electrode.

X-ray photoelectron spectroscopy (XPS) was used to characterize the chemical composition of the electrode surface. Figure 2 shows an overview of the XPS spectra for the undoped and Zr^4+^ doped TiO_2_ nanotube array electrodes. The Zr^4+^ doped electrode surface is mainly composed of Ti and O, containing a small amount of Zr (about 2.44 atom. %). The peak of C1s may be attributed to the contaminants on the sample surface. In addition, the binding energies of the peaks were calibrated by the binding energy of the C1s peaks (285 eV). The Zr 3D spectra (Figure 3a) show two obvious peaks, revealing that the Zr element was on the surface of the electrode. However, there was no diffraction peak of ZrO_2_ in the XRD pattern (Figure 1d), and the peak intensity of the TiO_2_ slightly decreased; its peak positions moved slightly to the right after the doping of Zr4+ (Figure 1e), indicating that the Zr should be incorporated into the TiO_2_ crystal lattice [20,21]. Figure 3b shows the deconvoluted XPS spectrum for the Ti 2p region. From the XPS-peak-differentiating analysis, it was found that, regardless of Zr^4+^-doping or not, Ti^3+^ and Ti^4+^ exist in the TiO_2_ electrodes. The four peaks correspond to the Ti^3+^ 2p3/2 (457.65 eV), Ti^4+^ 2p3/2 (458.95 eV), Ti^3+^ 2p1/2 (463.10 eV), and Ti^4+^ 2p1/2 (464.66 eV) [20]. However, for the undoped TiO_2_ nanotube array electrode, the Ti^3+^ content is very small (about 4.9 atom% of the total Ti). For the Zr^4+^-doped electrode, there is a significant increase in the area of two Ti^3+^ sub-peaks in Figure 3b, indicating an increase in the Ti^3+^ content (about 14.1 atom% of the total Ti). Compared with Figure 3c,d, the onset potential of the amorphous TiO_2_ nanotube array electrode for a hydrogen evolution reaction (HER) is significantly more negative than that of the pure titanium electrode, but no other redox peak was observed in the cyclic voltammetries (CVs) for both electrodes. However, for the undoped and Zr^4+^-doped anatase TiO_2_ nanotube array electrodes, there were nearly reversible redox peaks in the CVs, which corresponded to a transformation between Ti^4+^ and Ti^3+^ [22]. Moreover, after doping Zr^4+^, the oxidation peak current decreased, and reduction peak current increased, which indicated that it is beneficial to transform Ti^4+^ into Ti^3+^ on the anatase Zr/TiO_2_ nanotube array electrode. This is consistent with the previous XPS results.

**Figure 4:**
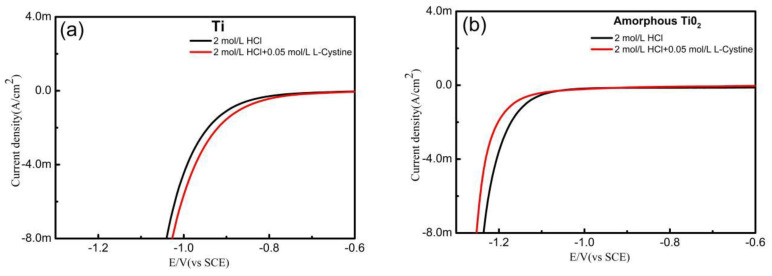

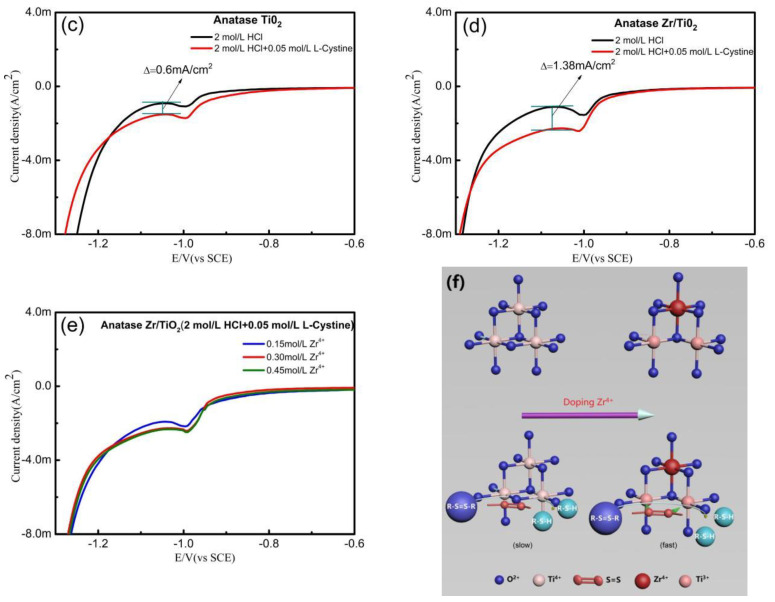
Linear sweep voltammetry (LSV) curves of different electrodes in 2 mol·L^−1^ HCl or 2 mol·L^−1^ HCl + 0.05 mol·L^−1^ L-cystine solutions (scan rate: 5 mV·s^−1^): (**a**) pure titanium, (**b**) amorphous TiO_2_ nanoarray tube, (**c**) anatase TiO_2_ nanoarray tube, and (**d**) anatase Zr/TiO_2_ nanotube array electrode; (**e**) the influence of concentrations of Zr(NO_3_)_4_ solution in chemical immersion process; (**f**) schematic illustration of electrocatalytic reduction mechanism of the anatase Zr/TiO_2_ nanotube array electrode for L-cystine.

Linear sweep voltammetry (LSV) was used to analyze the electrochemical behaviors for different electrodes in 2 mol·L^−1^ HCl solutions containing 0.05 mol·L^−1^ of L-cystine. For the pure Ti electrode (Figure 4a), no evident difference was observed in the LSV curves after adding the L-cystine into the HCl solution, and the hydrogen evolution reactions (HER) occurred at about 0.82V (vs. SCE). For the amorphous TiO_2_ nanotube array electrode, there was a similar tendency in the LSV curves, except that the HER potential was more negative than the pure Ti electrodes, as shown in Figure 4b. However, the hydrogen evolution reaction on the amorphous TiO_2_ nanotube array electrode seemed to be suppressed, to some extent, after adding the L-cystine. A possible reason for this is that the L-cystine can combine with H^+^ in the HCl solution, resulting in a decrease in the H^+^ concentration. For the undoped and Zr^4+^-doped anatase TiO_2_ nanotube array electrodes, the reduction of Ti^4+^ to Ti^3+^ was found during cathodic polarization, and the reduction current increased after doping Zr^4+^, as shown in Figure 4c,d. This indicates that the anatase structure of TiO_2_ is helpful in the formation of Ti^3^, and the dopant of Zr^4+^ can accelerate the transformation of Ti^4+^ to Ti^3+^. Moreover, when adding L-cystine to HCl solutions, the reduction currents increase in the two anatase TiO_2_ nanotube array electrodes, before hydrogen evolution is observed, which suggests that the anatase TiO_2_ possesses the electrocatalytic activities to reduce L-cystine. Compared with the undoped electrode, the reduction current on the Zr^4+^-doped electrode has a more obvious increase, and the maximum difference in current (1.38 mA cm^−2^) is about 2.26 times that of the undoped electrode (0.61 mA cm^−2^). These results prove that the Zr^4+^-doped TiO_2_ nanotube array electrode has good electrocatalytic reduction activity for reducing L-cystine. In order to illustrate the effect of the Zr dopant content, the electrocatalytic activity of the electrodes prepared in the different concentrations of Zr(NO_3_)_4_ solution during the chemical immersion process was studied using LSV, as shown in Figure 4e. From Figure 4e, the higher the concentrations of the Zr(NO_3_)_4_ solution, the higher the electrocatalytic reduction activity of the prepared electrode. This implies that the amount of Zr dopant increases with increasing concentrations of Zr(NO_3_)_4_ solution, from 0.15 to 0.30 mol·L^−1^. However, compared to the 0.30 mol·L^−1^ Zr(NO_3_)_4_ solution, the electrocatalytic reduction activity of the electrode prepared in the 0.45 mol·L^−1^ Zr(NO_3_)_4_ solution was not obviously improved. Figure 4f shows a schematic diagram of the catalytic reduction mechanism that reduces L-cystine on the Zr^4+^-doped TiO_2_ nanotube array electrode. Because Zr^4+^ has a similar d electron configuration and oxide structure to but larger ionic size than Ti^4+^, doping Zr^4+^ could not alter the crystalline structure of the anatase TiO_2_, but it did create the stress therein [15]. The strained effect induced the formation and enrichment of the adjacent bi-Ti^3+^, which also resulted in the increased oxygen vacancies. These are beneficial to the enhancement of active centers [15,16]. The Ti^3+^ ions have a stronger attraction to the S atom of L-cysteine, to induce the S=S bond to break down. Therefore, doping Zr^4+^ improves the electrocatalytic activity of the anatase TiO_2_ nanotube array electrode for the reduction of L-cystine.

L-cystine is not compatible with water, but it is easily soluble in acidic solutions. The reaction equation for the dissolution of its double sulfur bond structure in a HCl solution is [1]:

RSSR + 2H^+^ + 2Cl^−^→RSSR∙2HCl (R=CH_2_(NH_3_·HCl)COOH) (1)

The anatase TiO_2_ has oxygen vacancies and Ti^3+^ under a negative potential polarization. The reaction equation is [1]:

TiO_2_ + 4H^+^ + e^−^→Ti^3+^ + 2H_2_O (2)

Under the negative potential polarization, the Ti^3+^ reacts with the dissolved RSSR∙HCl in the solution, as follows:

2Ti^3+^ + RSSR∙2HCl + 2H^+^→2Ti^4+^ + 2RSH∙2HCl (3)

## 4. Conclusions

The Zr^4+^-doped anatase TiO_2_ nanotube array electrode was prepared through anodizing, combined with chemical immersion and heat treatment. Zr^4+^-doping into the anatase TiO_2_ induces the transformation of Ti^4+^ to Ti^3+^ and the formation of the oxygen vacancies, improving the electrocatalytic activity of the as-prepared electrode for L-cysteine reduction.
